# Lead Colic

**Published:** 1858-03

**Authors:** 


### Lead Colic.

Until within a few years past, this disease was regarded, by all, as having its seat in the digestive tube; the only difference of opinion between pathologists was in regard to the precise condition of this canal, and the cause of the pain. By some the pain was attributed to a distention of the bowel by gas, the result of constipation; by others, to the contact of the saturnine molecules with the mucous membrane. An occlusion of the intestine by an accumulation of hardened fœces has by others been regarded as the cause of all the pain; others, again, have attributed all to an inflammation of the intestine itself. From the character of the symptoms, however, some pathologists were induced to make farther researches which resulted in an entire change of their views as to the character of the disease—they regarding either the spinal cord or the spinal nerves and great sympathetic as the original seat of the affection. M. Briquet has recently read a paper upon the pathology, &c., of this disease, before the *Académie de Médecine*, in which he contends that the muscles of the walls of the abdomen are alone the seat of the pains in Lead Colic. The following extract from this paper in support of the above opinion, we find in the *Gazette Hebdomadaire* of the 15th Jan.

"1st. If we palpate the abdomen of a patient attacked with Lead Colic, so as to act alone upon the skin and subjacent muscles, or to use friction over the same, the patient experiences the most acute pain. 2d. The contraction, whether active or not, of the muscular fibres at the point where the pain is felt, increases greatly the sufferings of the patient, and in this way interferes with locomotion. 3d. Repose mitigates greatly the sufferings of the patient, and may alone entirely relieve the pain. 4th. The abdominal pains, in Lead Colic, are frequently accompanied either with hyperæsthesia or anæsthesia of the skin covering the painful muscles. The same thing may be observed in hysteria, in conditions where the superficial muscles are so frequently the seat of acute pain, the character of which is not often recognized. 5th. Constipation has no influence whatever over the abdominal pains. 6th. In some cases the abdominal walls are so very painful that there is set up such a state of contraction, as to cause a retraction of the abdomen. 7th. If by therapeutical means, of which we shall speak hereafter, we relieve suddenly and completely the pain produced by the compression of the sensitive muscle, all painful sensations cease at once.

Still, adds the author, I am far from pretending that the digestive tube is in a perfectly normal state in this disease. I

admit the existence of some intestinal derangement, but regard it of but little importance. I contend that in every case of colic, when not produced by the direct ingestion of lead by the stomach and bowels, the intestinal derangement is always secondary, and dependent upon other lesions, which in the treatment of the disease, demand our special attention.

